# Supplementary material for: Empirical methods for controlling false positives and estimating confidence in ChIP-Seq peaks
Source: BMC Bioinformatics. 2008 Dec 5;9:523. doi: 10.1186/1471-2105-9-523 (PMC2628906; doi:10.1186/1471-2105-9-523)
Supplement: Additional File 1 — A variety of html documents from the USeq web site detailing the available applications, their best usage, output file type descriptions, command line menus, etc. [file 1471-2105-9-523-S1.zip › USeqUserGuides/JavaDoc.zip!/JavaDoc/trans/anno/class-use/BindingRegion.html]

Uses of Class trans.anno.BindingRegion


|  |  |  |
| --- | --- | --- |
| **Overview** \| **Package** \| **Class** \| **Use** \| **Tree** \| **Deprecated** \| **Index** \| **Help** | |  |
| PREV   NEXT | **FRAMES**    **NO FRAMES**     **All Classes** |  |


---


## **Uses of Class trans.anno.BindingRegion**

| Packages that use BindingRegion | |
| --- | --- |
| **trans.anno** | For associating intervals, binding regions, and binding peaks with GFF annotation. |
| **util.bio.parsers** | Parsers and extractors, fasta, quality, text, gff.... |

Uses of BindingRegion in trans.anno

| Methods in trans.anno that return BindingRegion | |
| --- | --- |
| `static BindingRegion[]` | `IntersectRegions.fetchChromosomeSpecificBRs(BindingRegion[] sortedBRs, java.lang.String chromosome)`             Given a sorted array of BindingRegion and a chromosome, returns the appropriate BindingRegions. |
| `BindingRegion` | `BindingRegion.getClosestBindingRegion()` |
| `static BindingRegion[]` | `IntersectRegions.makeBindingRegions(int[][] regions, java.lang.String chromosome)`             Given an array of start stops and a chromosome, makes BindingRegions. |
| `static BindingRegion[]` | `AnnotateRegionsWithGeneList.makeRandomBindingRegions(BindingRegion[] br, java.util.HashMap chromLengths, int sizeNeighborhood)`             For each binding region this will make another binding region from the same chromosome with the same length, yet at a random location. |
| `static BindingRegion[]` | `AnnotateRegions.makeRandomBindingRegions(BindingRegion[] br, java.util.HashMap chromLengths, int sizeNeighborhood)`             For each binding region this will make another binding region from the same chromosome with the same length, yet at a random location. |
| `static BindingRegion[]` | `IntersectRegions.parseIntervalFile(java.io.File intervalFile)` |
| `static BindingRegion[]` | `AnnotateRegionsWithGeneList.parseIntervalFile(java.io.File intervalFile, int sizeNeighborhood)`             Attempts to fetch a serialized array of Interval[], then sorts/ ranks the intervals by the median ratio of the sub window. |
| `static BindingRegion[]` | `AnnotateRegions.parseIntervalFile(java.io.File intervalFile, int sizeNeighborhood)`             Attempts to fetch a serialized array of Interval[], then sorts/ ranks the intervals by the median ratio of the sub window. |
| `static BindingRegion[]` | `AnnotateRegionsWithGeneList.parsePicksFile(java.io.File picksFile, int sizeNeighborhood)` |
| `static BindingRegion[]` | `AnnotateRegions.parsePicksFile(java.io.File picksFile, int sizeNeighborhood)` |
| `static BindingRegion[]` | `IntersectRegions.parseRegionsFile(java.io.File picksFile)` |

| Methods in trans.anno with parameters of type BindingRegion | |
| --- | --- |
| `int` | `BindingRegion.bpIntersectionSameChromosome(BindingRegion other)`             Assumes regions are on the same chromosome, and the end base is included, not interbase numbering. |
| `static void` | `AnnotateRegionsWithGeneList.compareBindingRegionsVsGeneGrps(GeneGroup[] geneGroups, BindingRegion[] bindingRegions)`             Does a complete scan, could be optimized. |
| `static void` | `AnnotateRegions.compareBindingRegionsVsGeneGrps(GeneGroup[] geneGroups, BindingRegion[] bindingRegions)`             Does a complete scan, could be optimized. |
| `static boolean` | `IntersectRegions.containedWithin(BindingRegion one, BindingRegion two)`             Returns true if two entirely contains one. |
| `static int[]` | `AnnotateRegionsWithGeneList.countGenes(BindingRegion[] br)`             Returns: the number of genes with one or more binding regions within the neighborhood. |
| `static int[]` | `AnnotateRegions.countGenes(BindingRegion[] br)`             Returns the number of genes where the binding region is on the 5' end and the number of genes where the binding region is on the 3' end of the respective gene, the number of genes that overlap a binding region on their 5' end and 3' end, lastly the number of regions entirely contained by a gene, the number of binding regions with neighbors, the number of regions with no neighbors as defined by the neighborhood, the number of regions in non coding DNA, the number of regions in coding DNA, the number of regions that overlap coding and nonCoding DNA |
| `static int` | `AnnotateRegionsWithGeneList.countNumberBindingRegionsWithNeighbors(BindingRegion[] br)` |
| `static int` | `AnnotateRegions.countNumberBindingRegionsWithNeighbors(BindingRegion[] br)` |
| `static int` | `AnnotateRegionsWithGeneList.countNumberNeighbors(BindingRegion[] br)` |
| `static int` | `AnnotateRegions.countNumberNeighbors(BindingRegion[] br)` |
| `static BindingRegion[]` | `IntersectRegions.fetchChromosomeSpecificBRs(BindingRegion[] sortedBRs, java.lang.String chromosome)`             Given a sorted array of BindingRegion and a chromosome, returns the appropriate BindingRegions. |
| `static void` | `IntersectBindingPeaks.findClosestPeak(BindingRegion[] one, BindingRegion[] two)` |
| `static void` | `IntersectBindingPeaks.findClosestPeak(BindingRegion[] one, BindingRegion[] two)` |
| `int` | `AnnotateRegionsWithGeneList.findDistanceToATG(BindingRegion br, GeneGroup gp)`             Finds the distance to conservative estimate of an ATG, returns 0 if overlaps. |
| `int` | `AnnotateRegions.findDistanceToATG(BindingRegion br, GeneGroup gp)`             Finds the distance to conservative estimate of an ATG, returns 0 if overlaps. |
| `int` | `AnnotateRegionsWithGeneList.findDistToClosestATG(BindingRegion br)`             Finds the distance to the closest ATG translation start site. |
| `int` | `AnnotateRegions.findDistToClosestATG(BindingRegion br)`             Finds the distance to the closest ATG translation start site. |
| `int` | `AnnotateRegionsWithGeneList.findDistToClosestATG(BindingRegion br, java.util.ArrayList geneGroups)` |
| `int` | `AnnotateRegions.findDistToClosestATG(BindingRegion br, java.util.ArrayList geneGroups)` |
| `int` | `AnnotateRegionsWithGeneList.findDistToClosestTranscript(BindingRegion br)`             Finds the distance to the closest ATG translation start site. |
| `int` | `AnnotateRegions.findDistToClosestTranscript(BindingRegion br)`             Finds the distance to the closest ATG translation start site. |
| `int` | `AnnotateRegionsWithGeneList.findDistToClosestTranscript(BindingRegion br, java.util.ArrayList geneGroups)` |
| `int` | `AnnotateRegions.findDistToClosestTranscript(BindingRegion br, java.util.ArrayList geneGroups)` |
| `int` | `AnnotateRegionsWithGeneList.findDistToClosestTranscript(BindingRegion br, GeneGroup gp)`             Finds the distance to conservative estimate of start of first exon, returns 0 if overlaps. |
| `int` | `AnnotateRegions.findDistToClosestTranscript(BindingRegion br, GeneGroup gp)`             Finds the distance to conservative estimate of start of first exon, returns 0 if overlaps. |
| `void` | `IntersectRegions.intersectRandomBindingRegions(BindingRegion[] allTrialsOfSingleBR, BindingRegion[] chrOne)`             Assumes the same chromosome |
| `void` | `IntersectRegions.intersectRandomBindingRegions(BindingRegion[] allTrialsOfSingleBR, BindingRegion[] chrOne)`             Assumes the same chromosome |
| `int` | `BindingRegion.intersectReturnBP(BindingRegion other)`             Returns -1 for no overlap or the # bases of intersection. |
| `int` | `BindingRegion.intersectReturnGap(BindingRegion other)`             Returns -1 if on diff chromosomes, 0 if they overlap, or the # of bases between the two. |
| `boolean` | `BindingRegion.intersects(BindingRegion other)` |
| `static BindingRegion[]` | `AnnotateRegionsWithGeneList.makeRandomBindingRegions(BindingRegion[] br, java.util.HashMap chromLengths, int sizeNeighborhood)`             For each binding region this will make another binding region from the same chromosome with the same length, yet at a random location. |
| `static BindingRegion[]` | `AnnotateRegions.makeRandomBindingRegions(BindingRegion[] br, java.util.HashMap chromLengths, int sizeNeighborhood)`             For each binding region this will make another binding region from the same chromosome with the same length, yet at a random location. |
| `static int` | `IntersectRegions.numberBases(BindingRegion[] brs)` |
| `int` | `BindingRegion.overlap(BindingRegion other)`             Returns -1 for no overlap, 0 for complete overlap, or a positive int for the # bases of overlap. |
| `void` | `AnnotateRegionsWithGeneList.printDistToClosestATGAndTranscript(BindingRegion[] br)`             Prints rank, chrom, start, stop, distance to closest ATG, to closest transcript start. |
| `void` | `AnnotateRegions.printDistToClosestATGAndTranscript(BindingRegion[] br)`             Prints rank, chrom, start, stop, distance to closest ATG, to closest transcript start. |
| `void` | `BindingRegion.setClosestBindingRegion(BindingRegion closestBindingRegion)` |
| `static double[]` | `IntersectRegions.statBindingRegionArray(BindingRegion[] brs)` |
| `static boolean` | `IntersectRegions.writeBindingRegions(BindingRegion[] brs, java.io.File file)` |

Uses of BindingRegion in util.bio.parsers

| Methods in util.bio.parsers with parameters of type BindingRegion | |
| --- | --- |
| `static int` | `FetchGenomicSequences.numberBases(BindingRegion[] brs)` |
| `static double[]` | `FetchGenomicSequences.statBindingRegionArray(BindingRegion[] brs)` |

---


|  |  |  |
| --- | --- | --- |
| **Overview** \| **Package** \| **Class** \| **Use** \| **Tree** \| **Deprecated** \| **Index** \| **Help** | |  |
| PREV   NEXT | **FRAMES**    **NO FRAMES**     **All Classes** |  |


---
